# Supplementary material for: Cerebral Microvascular Perfusion Assessed in Elderly Adults by Spin-Echo Dynamic Susceptibility Contrast MRI at 7 Tesla
Source: Tomography. 2024 Jan 18;10(1):181–92. doi: 10.3390/tomography10010014 (PMC10819808; doi:10.3390/tomography10010014)
Supplement: Supplementary file 1 [file tomography-10-00014-s001.zip › tomography-2721499-supplementary.pdf]

Table S1: Sensitivity analysis investigating the effect of large vessel exclusion on the perfusion results.

A one-sample t-test was performed to test whether the CBF ratios between CGM and WM were significantly different, after 0%, 10%, 20%, 30%, 40% or 50% of the largest vessels were excluded from the analysis. The results are shown below (P<0.05 was assumed to be significant).

0% largest vessel exclusion

**CBF in**	**CGM**		**WM**	
**Control variables**	**Std. β**	**P-value**	**Std. β**	**P-value**
**Age**	-0.299	0.042*	-0.114	0.457
**Sex**	0.417	0.006*	0.348	0.028*

10% largest vessel exclusion

**CBF in**	**CGM**		**WM**	
**Control variables**	**Std. β**	**P-value**	**Std. β**	**P-value**
**Age**	-0.304	0.037*	-0.151	0.321
**Sex**	0.428	0.004*	0.366	0.020*

20% largest vessel exclusion

**CBF in**	**CGM**		**WM**	
**Control variables**	**Std. β**	**P-value**	**Std. β**	**P-value**
**Age**	-0.308	0.033*	-0.167	0.266
**Sex**	0.448	0.003*	0.395	0.011*

30% largest vessel exclusion

**CBF in**	**CGM**		**WM**	
**Control variables**	**Std. β**	**P-value**	**Std. β**	**P-value**
**Age**	-0.322	0.024*	-0.177	0.233
**Sex**	0.461	0.002*	0.424	0.006*

40% largest vessel exclusion

CBF in	CGM		WM	
**Control variables**	**Std. β**	**P-value**	**Std. β**	**P-value**
**Age**	-0.331	0.019*	-0.194	0.184
**Sex**	0.479	0.001*	0.445	0.004*

50% largest vessel exclusion

CBF in	CGM		WM	
**Control variables**	**Std. β**	**P-value**	**Std. β**	**P-value**
**Age**	-0.355	0.012*	-0.215	0.134
**Sex**	0.474	0.001*	0.479	0.002*
